# Consultation diagnoses and procedures billed among recent graduates practicing general otolaryngology – head & neck surgery in Ontario, Canada

**DOI:** 10.1186/s40463-018-0293-8

**Published:** 2018-07-20

**Authors:** Antoine Eskander, Paolo Campisi, Ian J. Witterick, David D. Pothier

**Affiliations:** 1 Department of Otolaryngology - Head & Neck Surgery, Surgical Oncology, University of Toronto, Sunnybrook Health Sciences Centre and Michael Garron Hospital, Toronto, ON Canada; 2 Institute for Clinical Evaluative Sciences (ICES), Toronto, ON Canada; 3 Department of Otolaryngology – Head & Neck Surgery, Sunnybrook Health Sciences Centre and the Odette Cancer Centre, Michael Garron Hospital, Endocrine Surgery, 2075 Bayview Ave., M1-102, Toronto, ON M4N 3M5 Canada; 4 Department of Otolaryngology – Head & Neck Surgery, Hospital for Sick Children, Toronto, ON Canada; 5 Department of Otolaryngology – Head & Neck Surgery, Mount Sinai Hospital, Sinai Health System, Toronto, ON Canada; 6 Department of Otolaryngology – Head & Neck Surgery, Toronto General Hospital, University Health Network, Toronto, ON Canada

**Keywords:** Medical education, Consultation, Diagnoses, Procedures, Volume, Recent graduates, Otolaryngology

## Abstract

**Background:**

An analysis of the scope of practice of recent Otolaryngology – Head and Neck Surgery (OHNS) graduates working as general otolaryngologists has not been previously performed. As Canadian OHNS residency programs implement competency-based training strategies, this data may be used to align residency curricula with the clinical and surgical practice of recent graduates.

**Methods:**

Ontario billing data were used to identify the most common diagnostic and procedure codes used by general otolaryngologists issued a billing number between 2006 and 2012. The codes were categorized by OHNS subspecialty. Practitioners with a narrow range of procedure codes or a high rate of complex procedure codes, were deemed subspecialists and therefore excluded.

**Results:**

There were 108 recent graduates in a general practice identified. The most common diagnostic codes assigned to consultation billings were categorized as ‘otology’ (42%), ‘general otolaryngology’ (35%), ‘rhinology’ (17%) and ‘head and neck’ (4%). The most common procedure codes were categorized as ‘general otolaryngology’ (45%), ‘otology’ (23%), ‘head and neck’ (13%) and ‘rhinology’ (9%). The top 5 procedures were nasolaryngoscopy, ear microdebridement, myringotomy with insertion of ventilation tube, tonsillectomy, and turbinate reduction. Although otology encompassed a large proportion of procedures billed, tympanoplasty and mastoidectomy were surprisingly uncommon.

**Conclusion:**

This is the first study to analyze the nature of the clinical and surgical cases managed by recent OHNS graduates. The findings demonstrated a prominent representation of ‘otology’, ‘general’ and ‘rhinology’ based consultation diagnoses and procedures. The data derived from the study needs to be considered as residency curricula are modified to satisfy competency-based requirements.

## Background

The Royal College of Physicians and Surgeons of Canada (RCPSC) is in the process of implementing competency based medical education (CBME) across all medical and surgical specialties. CBME is fundamentally based on the acquisition of specific competencies called entrustable professional activities (EPAs) . [1] The specific type of CBME used by the RCPSC is called Competency by Design (CBD). Otolaryngology - Head & Neck Surgery (OHNS) is one of the specialties at the forefront of CBD implementation. As such, OHNS needs to align its training programs to the competencies that are required for a primary otolaryngology practice in preparation for the human resource demands of the country. [2, 3]

Another factor that may influence CBD is the changing OHNS work force in Canada with an increase in medical or non-surgical OHNS and an increase in office-based procedures which is due, at least in part, to a lack of operating room availability and increasingly subspecialized care at high volume centres. [4] An analysis of the scope of practice of recent OHNS graduates working as general otolaryngologists has not been previously performed. Data derived from this study will be a useful guide in determining the scope of the required Entrustable Professional Activities (EPA).

The objective of this study is to assess consultation diagnoses and procedures billed among recent graduates practicing general OHNS in Ontario, Canada. The ultimate aim is to provide data to clinician educators as they consider restructuring OHNS training in Canada through CBD. It is hypothesized that general procedures (myringotomy and tubes, adenotonsillectomy, septoplasty and turbinate reduction) occupy the majority of the surgical volume while medical otologic diagnoses occupy a significant portion of the clinical volume of new OHNS physicians in Ontario.

## Methods

### Data acquisition - Ontario healthcare

The data for the study were requested from the Ontario Ministry of Health and Long Term Care (MOHLTC) in an anonymized and aggregated format lacking patient level information such as age and gender from the Claims History Database. Aggregations were unique on physician identification number, and a unique combination of billing and diagnosis codes organized by billing year with frequency of services provided for each combination.

### Subspecialty coding of all diagnosis and billing codes

For each billing code, either for consultation or procedures, physicians must submit an associated diagnosis code based on the International Statistical Classification of Disease and Related Health Problems (ICD-9) diagnosis coding system. Two co-authors (A.E., I.W.) reviewed each unique vcombination of diagnosis code with billing code to assign a subspecialty within OHNS; General/Laryngology, Facial Plastic and Reconstructive Surgery, Pediatric, Endocrine, Head and Neck Oncology, Rhinology, and Otology. This was an iterative process and disagreements were resolved by consensus among all co-authors. Of note, some diagnostic codes were a priori thought to potentially belong in more than one category and certain categories were combined (e.g. General and Laryngology). Furthermore, during our data presentation, the top diagnostic codes are presented for each subspecialty to allow for transparency.

Given that the physician level data did not include a patient age identifier, the dataset inherently underestimates the specific pediatric consultation and procedure codes. However, under general procedures, a special subgroup was extracted specifically addressing myringotomy and tubes as well as tonsillectomy and adenoidectomy (M&T T&A) given that these are the most common pediatric procedures in OHNS. Non-otolaryngological procedures which were deemed to be entered by error were excluded from the analysis. Procedures which may have been performed in the clinic setting and those that required an operating room were not treated differently given our study question and primary objective. Rather, consultation code diagnoses and any procedural or technical skills were treated separately given that these are typically assessed separately in CBD.

### Exclusion criteria - subspecialist physicians

Procedure and consultation codes by subspecialty were then summarized to identify and exclude subspecialists. This is important given the objective of the study is to identify recent graduates who practice general OHNS. However, consultation codes with their associated diagnosis codes were found to be not specific to subspecialists. Therefore, procedure codes were used to asses each physician individually with regards to the percentage of their procedures performed in each subspecialty. A decision rule was used to identify potential subspecialist practices; when more than 50% of the procedures fell within one subspecialty, the procedures performed for that physician within that subspecialty were then explored prior to determining whether they should be excluded from the analysis. Having greater than 50% of procedure codes within one subspecialty did not necessarily mean exclusion as some were not deemed to be subspecialist procedure codes. Consensus was then achieved amongst the co-authors as to which physicians should be excluded secondary to a subspecialist procedure billing pattern. It should be noted, that there was a very clear and easy to identify procedure billing pattern between generalists and subspecialists using this methodology. General OHNS who were performing largely cosmetic procedures would not have been excluded as these procedures are not captured in the billing and would have been grouped amongst generalists. More specifically, in our healthcare jurisdiction, cosmetic procedures are not covered under the provincial health care insurance plan and are billed privately, therefore, these are not captured.

### Analysis

Using all clinical encounters, we assessed the clinical non-operative breadth and volume of recent OHNS graduates. These included audiological professional but not technical fees under ‘otology’. We then narrowed our assessment to new consultations as a separate analysis which excluded follow-up visits and audiological professional fee codes. To do each of these, we used a mean of means methods; every physician had an average percentage of clinical encounters in each subspecialty which were averaged amongst the total physician population. The range of ICD-9 diagnostic codes was assessed by subspecialty. The top 10 codes in each subspecialty were assessed as a proportion of the subspecialty for the consultation codes only.

Similarly, procedure codes were assessed for breadth and volume by subspecialty. A similar mean of means method was used. We excluded B suffix procedure codes because these are assistant surgeon codes and in our dataset were most often used by very low volume head and neck surgeons, who were likely clinical fellows in the dataset. These were excluded when we were narrowing our cohort of physicians to generalists. We then assessed the top 15 procedures performed by OHNS followed by the top 10 procedures in each subspecialty as a proportion of the subspecialty. Data are presented in a manner so that reverse calculations can be performed to allow increased granularity. This also allows for disagreements on subspecialty of procedure to be resolved for the top procedures. All data manipulation and descriptive statistics were performed using SAS version 9.4 (SAS Institute, Cary, NC).

## Results

### Description of cohort and exclusions

The original file provided by the MOHLTC had a total of 383,928 rows with complete billing data for 6 fiscal years (April 1, 2006 to March 31, 2012) for 129 physicians who had acquired new physician billing numbers within the prior 10 years (2003–2012). Each row could represent multiple billing encounters with a frequency provided for the fiscal year. Each physician had a varying amount of data contributing to the dataset depending on the number of fiscal years for which they had a billing number.

**Fig. 1:**
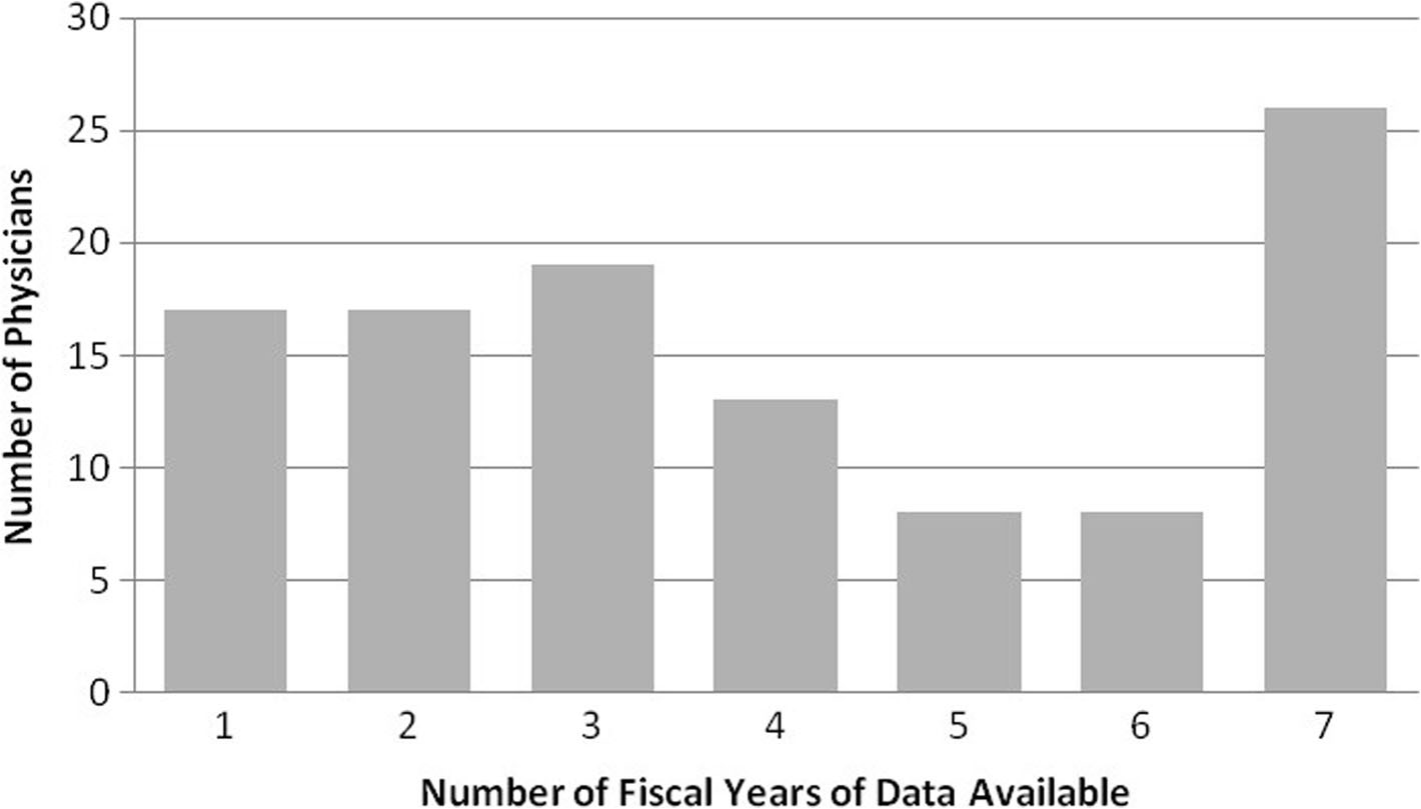
Number of Physicians and Fiscal Years in Practice For Surgeons (*n* = 108; 2006–2012)

Using the 50% procedure within a single subspecialty rule followed by exploration of the procedure codes within the dominant subspecialty, we excluded 21 physicians. Three surgeons performed endocrine surgery nearly exclusively, 3 performed mostly facial plastic surgery procedures in low volume and were assumed to be facial plastic surgeons, 6 performed advanced head and neck oncologic procedures, 1 performed sinus surgery almost exclusively, 1 physician practiced sleep medicine, and 7 practiced advanced otological procedures. This left us with a final cohort of 108 physicians. We had an average of 3.98 years (2.19 standard deviation) of data per physician (Figure 1).

### Clinic volume

Using all clinical encounters, we assessed the clinical non-operative breadth and volume of recent OHNS graduates (Table 1). These included audiological professional but not technical fees under otology leading to a majority (61.6%) of this clinical volume falling under otology followed by general OTL (22.0%) and rhinology (9.6%).

**Table 1:** Summary Non-Procedural/Non-Operative Clinic Volume

*Subspecialty*	Average %*	Average %**
Otology	61.6	42.3
General/Laryngology	22.2	35.0
Rhinology	9.6	17.2
Head and Neck	3.4	2.6
Endocrine	2.2	1.7
Facial Plastics	0.5	0.7
Pediatric	0.5	0.5

*includes audiological professional billing codes and follow up visits

**excludes audiological professional fees and follow up visits; focuses exclusively on new consultations

When audiological professional billing codes and follow up visits were excluded, and only new consultations are assessed by diagnosis code (Table 1), otology continues to be the most common (42.3%) subspecialty seen in consultation by recent OHNS graduates followed by general (35%) and rhinology (17.2%).

### Consultation diagnosis codes by subspecialty

Table 2 highlights the range of diagnosis codes in each subspecialty seen by recent OHNS generalists in our cohort. The general category had the most diagnosis codes followed by head and neck and endocrine. Facial plastic surgery had the least number of diagnosis codes, with only 3, all of which were facial fracture codes.

**Table 2:** Number of Diagnosis Codes per subspecialty

*Subspecialty*	*Number of ICD-9 Diagnosis Codes*
General	85
Head and Neck	22
Endocrine	13
Pediatric	10
Otology	10
Rhinology	6
Facial Plastics	3

The top consultation diagnosis codes by subspecialty are outline in Table 3. The top diagnoses in otology include deafness, wax or cerumen in ear, and serous otitis media. Epistaxis, hypertrophy or chronic infection of tonsils and/or adenoids, and acute laryngitis were the top diagnosis codes in the general category. The top rhinologic diagnoses in consultation were allergic rhinitis, deviated nasal septum and chronic sinusitis. The top generalist head and neck diagnoses seen in consultation were non-melanoma skin cancers, laryngeal and tongue cancers. The most common endocrine diagnoses were thyroiditis, thyroid neoplasm, and goiter. There were no specific pediatric diagnoses in consultation given that we could not determine patient age and none of the ICD-9 codes were pediatric/age-specific.

**Table 3:** Top Diagnosis Codes (ICD 9) in consultation organized by subspecialty

Rank	ICD-9	Otology (42.3%)	%	ICD-9	General (35.0%)	%
1	389	Deafness	29.9	786	Signs/Symptoms not yet diagnosed - Respiratory System - Epistaxis, Hemoptysis	25.1
2	388	Wax or cerumen in ear, other disorders of ear and mastoid, tinnitus	20.3	474	Hypertrophy or Chronic Infection of Tonsils and/or Adenoids	17.6
3	381	Serous otitis media, eustachian tube disorders	19.5	464	Acute Laryngitis, Tracheitis, Croup, Epiglottis	14.8
4	780	Signs/symptoms not yet diagnosed - nervous system - Convulsions, Ataxia	14.5	463	Acute Tonsillitis	5.8
5	380	Otitis Externa	6.9	226	Benign Neoplasms - Thyroid e.g. Adenoma or Cystadenoma	4.4
6	386	Meniere’s Disease, Labyrinthitis	2.9	527	Disease of Salivary Glands	4.0
7	382	Suppurative Otitis Media	2.5	210	Benign Neoplasms - Lip, Oral Cavity, Pharynx	3.4
8	384	Perforation of Tympanic Membrane	2.5	529	Glossitis	2.5
9	387	Otosclerosis	0.6	528	Stomatitis, Aphthous Ulcers	2.5
10	383	Mastoiditis	0.3	530	Esophagitis, Cardiospasm, Ulcer of Esophagus, Stricture, Stenosis	2.0
Rank	ICD-9	Rhinology (17.2%)	%	ICD-9	Facial Plastics (0.7%)	
1	477	Allergic Rhinitis, Hay Fever	28.4	802	Fractures and Fracture/Dislocations - Facial Bones	93.5
2	470	Deviated Nasal Septum	24.4	829	Fractures and Fracture/Dislocations - all other fractures	3.5
3	473	Chronic Sinusitis	21.7	803	Fractures and Fracture/Dislocations - Skull	3.0
4	461	Acute Sinusitis	14.1			
5	471	Nasal Polyp	10.4			
6	160	Nasal Cavities, Middle Ear and Accessory Sinuses	1.0			
Rank	ICD-9	Head and Neck (2.6%)	%	ICD-9	Endocrine (1.7%)	%
1	173	Other skin malignancies	58.4	245	Thyroiditis	27.8
2	161	Malignant Neoplasms - Larynx, Trachea	16.1	193	Malignant Neoplasms - Thyroid	22.0
3	141	Malignant Neoplasms -Tongue	8.7	241	Nontoxic Nodular Goitre	21.2
4	202	Other Malignant Neoplasms	3.6	240	Simple Thyroid Goiter	14.0
5	239	Unspecified Neoplasms e.g. Polycythemia Vera	2.6	252	Parathyroid Gland Disorders	5.1
6	172	Malignant Neoplasms - Melanoma of Skin	2.3	242	Hyperthyroidism, Thyrotoxicosis, Exophthalmic Goitre	4.2
7	142	Major Salivary Glands	1.9	237	Endocrine Glands and Nervous System	2.7
8	140	Malignant Neoplasms - Lip	1.2	259	Other Endocrine Disorders	1.5
9	196	Secondary Neoplasm of Lymph Nodes	1.2	253	Pituitary Gland Disorders	0.9
10	200	Malignant Neoplasms - Lymphosarcoma, Recticulum Cell Sarcoma	0.9	227	Other Endocrine Glands and Related Structures	0.3

### Top procedure codes

The top procedural codes, regardless of subspecialty demonstrate a high volume of fiberoptic examination (35%), ear debridement for cerumen and debridement of mastoid cavities under the microscope (11 and 8% respectively), and myringotomy with insertion of ventilation tubes (3%). These procedures alone comprise (57%) of all procedural codes performed by recent OHNS graduates in Ontario. The remaining procedures on the list include tonsillectomy, turbinate reduction, total thyroidectomy, cautery for epistaxis, nasal polypectomy and rigid endoscopic examination of the airway. Intranasal ethmoidectomy including maxillary antrostomy with endoscope (M083 + E844) did not achieve top 10 status given these are two separate codes but if these codes were to be combined it would rank between tonsillectomy (rank 5) and turbinate reduction (rank 6) in Table 4.

**Table 4:** Top 15 Overall Procedure

Rank	Code	Description	Subspecialty	%
1	Z296	Fiberoptic endoscopy of upper airway with flexible endoscope	General	35
2	G420	Ear syringing, curetting or debridement	Otology	11
3	Z907	Debridement under microscopy, debridement of mastoid cavities, and/or ears with significant external or middle ear pathology but not for removal of cerumen - unilateral	Otology	8
4	Z914	Myringotomy with insertion ventilation tube	General (M&T T&A)	3
5	S063	Tonsillectomy	General (M&T T&A)	2
6	Z302	Turbinate Reduction	Rhinology	1
7	S788	Total Thyroidectomy	Endocrine	1
8	Z314	Chemical/electrocautery for epistaxis	Rhinology	1
9	Z305	Nasal polypectomy - general anesthesia	Rhinology	1
10	Z299	Fiberoptic endoscopy of upper airway with rigid endoscope	General	1
11	M083 E844	Intranasal ethmoidectomy incl maxillary antrostomy with endoscope	Rhinology	1
12	M012	Septoplasty	Rhinology	1
13	Z771	Thyroid Fine Needle Aspiration	Endocrine	1
14	Z118	Superficial Lump Fine Needle Aspiration	General	< 1
15	Z502	Excision Mouth Lesion - less than 2 cm.	Head and Neck	< 1

### Top procedure codes by subspecialty

**Fig. 2:**
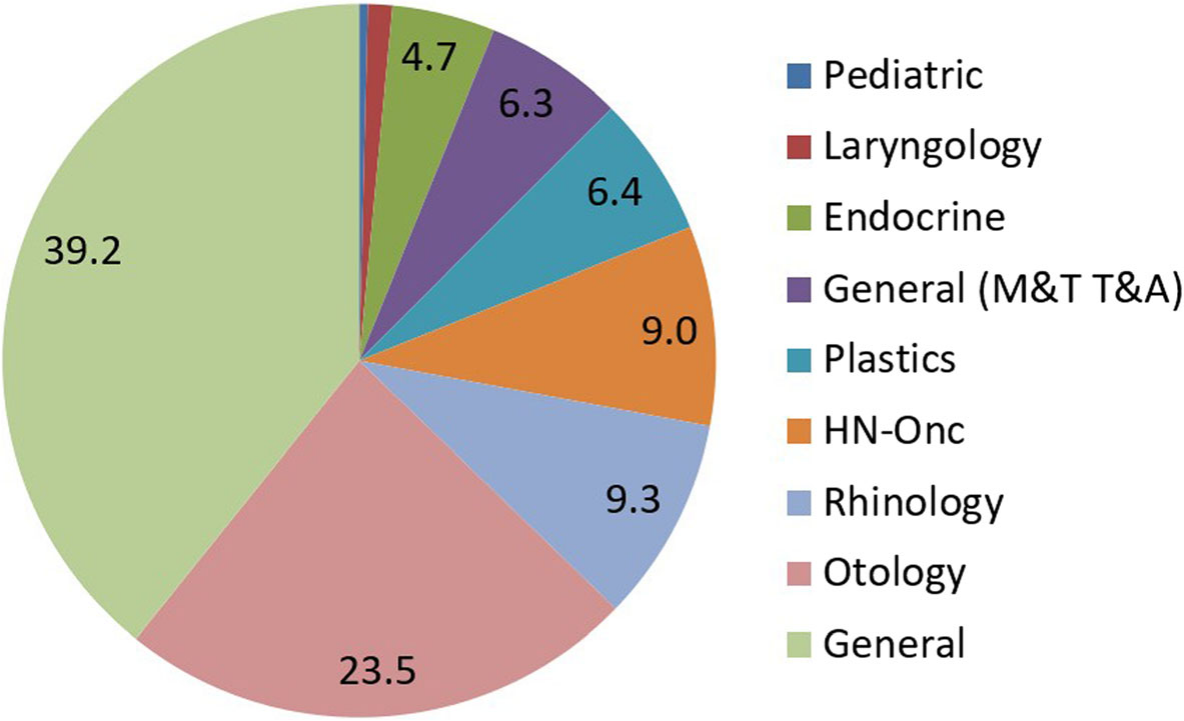
Summary of Procedural/Operative Volume

The most common procedures fall under general OHNS (39.2%) followed by otology (23.5%), and rhinology (9.3%). However, if the category which includes myringotomy/tubes and tonsils/adenoids is combined with general, and if endocrine is combined with head and neck the order changes (Table 5). General (including the most common pediatric procedures - myringotomy/tubes and tonsils/adenoids) remains the most common set of procedures performed (45.5%) followed by otology (23.5) and then head and neck when it includes endocrine surgery (13.7%) (Figure 2).

**Table 5:** Summary of Procedural/Operative Volume

*Subspecialty*	*%*
General	39.2
Otology	23.5
Rhinology	9.3
Head and Neck	9.0
Facial Plastics	6.4
General (M&T T&A)	6.3
Endocrine	4.7
Laryngology	1.1
Pediatric	0.4

The most common procedures by subspecialty are presented in Table 6. General procedures are dominated by flexible fiberoptic examination of the upper airway (82%). Myringotomy with ventilation tubes and tonsillectomy comprise 57 and 29% respectively of the separate general (M&T T&A) category. The most common otolgic procedures include ear debridement for cerumen and debridement of mastoid cavities under the microscope (54 and 37% respectively), followed by type one tympanoplasty (1%) and myringoplasty (1%). Of note, advanced otologic procedures such as stapedotomy/stapedectomy, mastoidectomy, complex tympanoplasty requiring ossiculoplasty or mastoidectomy, do not comprise any of the top 10 otologic codes.

**Table 6:** Top Procedure Codes by Subspecialty

Rank	Code	General (39.2%)	%	Code	Otology (23.5%)	%
1	Z296	Fiberoptic endoscopy of upper airway with flexible endoscope	82	G420	Ear syringing, curetting or debridement	54
2	Z299	Fiberoptic endoscopy of upper airway with rigid endoscope	2	Z907	Debridement under microscopy, debridement of mastoid cavities, and/or ears with significant external or middle ear pathology but not for removal of cerumen - unilateral	37
3	Z118	Aspiration of superficial lump for cytology	2	E336	Tympanoplasty-type one	1
4	Z915	Removal foreign body (simple)	1	E323	Myringoplasty	1
5	Z501	Mouth Incisional Biopsy	1	E301	Resection of pinna - with local flap	< 1
6	Z326	Change of tracheostomy tube	1	E300	Resection of pinna - with primary closure	< 1
7	Z515	Oesophagoscopy, with or without biopsy(ies)	1	Z908	Debridement under microscopy, debridement of mastoid cavities, and/or ears with significant external or middle ear pathology but not for removal of cerumen - unilateral; General Anesthetic	< 1
8	Z413	Scalene node fine needle aspiration	1	Z904	Local Excision of Polyp of external ear (office)	< 1
9	Z327	Flexible or rigid bronchoscopy, with or without bronchial biopsy or suction	< 1	Z866	Removal of Ear Foreign Body (complicated) under general anesthetic	< 1
10	Z116	Skin Incisional Biopsy	< 1	Z916	Intratympanic Injection	< 1
Rank	Code	Head Neck (9.0%)	%	Code	Endocrine (4.7%)	%
1	R915	Comprehensive dissection of neck lymph nodes, must include 3 or more levels - unilateral	43	S788	Total Thyroidectomy	33
2	Z502	Excision Mouth Lesion - less than 2 cm.	6	S789	Subtotal Thyroidectomy	25
3	Z355	Quadroscopy or panendoscopy	3	Z771	Aspiration biopsy, thyroid gland/nodule fine needle method	18
4	R048	Simple Excision of Face or Neck Lesion - single	3	S795	Exploration and/or removal, parathyroids or parathyroid tumour	6
5	R910	Limited neck dissection, must include 2 levels (unilateral) or central compartment	3	E880	Parathyroid Reimplantation	3
6	M106	Excision of Mediastinal Tumor	3	S793	Completion Thyroidectomy	3
7	S043	Total Parotidectomy with facial nerve preservation	2	S790	Hemi-thryoidectomy	2
8	S003	Excision Mouth Lesion - 2 - 4 cm.	2	S061	Thyroglossal Duct Remnant Excision	2
9	E540	Skin Malignant Lesion wide excision in any area and must include > 1 cm margins and layered closure - performed in hospital with frozen section	2	S792	Re-exploration of neck for hyperparathyroidism	< 1
10	Z119	Cryotherapy treatment of multiple pre-malig actinic keratosis	1	S787	Excisional Surgical Biopsy of the Thyroid Gland	< 1
Rank	Code	Rhinology (9.3%)	%	Code	General- (T&A M&T) (6.3%)	%
1	Z302	Turbinate Reduction	13	Z914	Myringotomy with insertion of ventilation tube	57
2	M083 E844	Intranasal ethmoidectomy incl maxillary antrostomy with endoscope	11	S063	Tonsillectomy	29
3	Z314	Chemical/electrocautery for epistaxis	10	Z912	Myringotomy	8
4	Z305	Nasal polypectomy - general anesthesia	10	S065	Adenoidectomy	7
5	M012	Septoplasty	8			
6	M054	Intranasal maxillary antrostomy – unilateral – by endoscopic or endonasal approach.	6			
7	Z315	Epistaxis - anterior packing	4			
8	Z318	Trephine or endoscopic frontal sinusotomy	4			
9	Z311	Removal of nasal foreign body (simple)	3			
10	M060	Ethmoidectomy-intranasal-unilateral	2			
Rank	Code	Facial Plastics (6.4%)	%	Code	Laryngology (1.1%)	%
1	R046	Rotations, Transpositions, Z-plasties of the face, neck, or scalp defect 2.1 cm to 5 cm	10	Z321	Direct Laryngoscopy-with or without biopsy	37
2	F136	Nasal Bones Closed Reduction	6	E600	Laryngoscopy using operative microscope	20
3	R045	Rotations, Transpositions, Z-plasties of the face, neck, or scalp defect less than 2 cm	5	Z323	Direct Laryngoscopy-with removal lesions	19
4	R011	Advancement flaps of the face, neck, scalp defect 2.1 cm to 5 cm	4	E643	Direct Laryngoscopy when using laser with microlaryngoscopy for benign disease	4
5	Z122	Excision of cyst, hemangioma, lipoma of face or neck	3	Z320	Insertion of voice prosthesis	4
6	R022	Scar revision of face or neck defect 2.6 cm–5 cm.	3	Z346	Transtracheal aspiration	2
7	R087	Split thickness graft - major - complex area	2	M090	Laryngoplasty	2
8	E551	Bone, fascial or dermal grafts - autogenous - separate incision	2	M080	Teflon augmentation larynx	2
9	G396	Injection of extensive keloids	2	G873	Botulinum toxin injection(s) for spasmodic dysphonia	1
10	R012	Advancement flaps of the face, neck, scalp defect 5.1 cm to 10 cm	2	M085	Arytenoidectomy or arytenoidopexy or lateralization procedure	1
Code	Pediatrics (0.4%)					%
E622	Any bronchoscopic procedure for patient under 3 years of age					79
R043	Removal of congenital dermoid cyst in an infant or child					3

Common head and neck procedures include comprehensive and selective neck dissections (43 and 3% respectively), excision of oral cavity lesions (8%), excision of skin lesions (5%), quadroscopy (3%), and parotidectomy (2%). Endocrine procedures total and subtotal/hemi-thyroidectomy (33 and 27% respectively), fine needle aspiration biopsy (18%), and parathyroid related procedures (9%). Rhinological procedures include turbinate reduction (13%), maxillary antrostomy and ethmoidectomy (11%), cautery for epistaxis (10%), nasal polypectomy (10%) and septoplasty (8%). Many of these procedures are performed endoscopically based on the fee schedule coding and common practice. Facial plastic surgery procedures included rotational flaps (15%), advancement flaps (6%), and reduction of nasal bones (6%). Other facial trauma procedures did not make the top 10 list for facial plastic surgery in Ontario. Laryngological procedures included direct laryngoscopy with or without biopsy (37%), laryngoscopy with use of operative microscope (20%), laryngoscopy with removal of lesions (19%), and laser microlaryngoscopy (4%).

## Discussion

As OHNS integrates CBD into its training programs, it is an opportunity to assess and modify required training experiences. To do this logically and systematically, programs need to understand the needs of the ‘new’ graduate and accurately monitor the training experiences of current residents through clinical encounter and operative logs. This paper focuses on understanding the nature of practices of recent graduates.

Our study demonstrates that otologic consultation diagnoses are the most common (61.6% if audiological assessment is included and 42.3% if audiological assessment is not included) among recent OHNS graduates. This is followed by general (35%) and rhinologic (17.2%) diagnoses. The most common otologic diagnoses are deafness, cerumen impaction, and serous otitis media. Procedural volume demonstrated a different trend with general (45.52% when M&T T&A is included) dominating, followed by otologic procedures (23.5%). Despite head and neck oncology and endocrine consultations representing only 5.6% of consultations, it disproportionately impacted procedure volume occupying 13.7% (ranking third) of the procedures performed.

There is no previous literature assessing the clinical and procedural tasks performed by recent OHNS graduates using either individual program data or population-based data as was performed in this study. Despite our preliminary results, individual training program clinical and procedure volume by subspecialty would need to be assessed prior to making any adjustments to residency curricula. Programs are likely to use resident case logs to assess training volume. Unfortunately, case logs are notoriously inaccurate largely due to underreporting of cases performed despite program directors advising residents to log all procedures. [5] This is particularly true for procedures performed outside of the operating room and common procedures, both of which dominated our results. [5] Case logging methods also vary greatly between programs. Moving forward with a national CBD curriculum, case logging will be standardized and submitted prior to graduation from residency to the Royal College of Physicians and Surgeons of Canada (RCPSC). [5] Surgical case numbers vary drastically between programs, and despite new minimum volume of key indicator procedures recommended by the Accreditation Council for Graduate Medical Education (ACGME) in the United States (U.S.), many graduating residents have not met these requirements. [6] Anecdotally, we know the same to be true for Canadian programs.

Even with adequate case logs, there is disagreement among program directors and residents regarding the minimum number of procedures required to achieve competence in these procedures. In one study comparing general surgery (GS) and otolaryngology (OHNS) residents with regards to obtaining competency in thyroid surgery, residents believed that only 13 and 25 (GS/OHNS) thyroidectomies were required by their respective boards prior to graduation and both groups felt that 30 (27/33) thyroid operations were necessary to obtain competence (*p* < .01). [7] This demonstrates that board requirements and the perceived number of operations required for CBD may differ by specialty. Unfortunately, most residents who responded (average post-graduate year 3.5) had completed only between 1 and 10 thyroidectomies. Similarly, tracheotomy, a procedure which did not make it onto any of the top 10 lists in any of the subspecialties in our study, is declining among otolaryngology training programs and increasing in general surgery training programs, however, current OHNS trainees continue to perform more of these procedures by the end of training than their GS counterparts. [8]

With regards to tracheotomy, and other key indicator procedures, suggestions that less training is required in this domain because it is not frequently performed by recent OHNS graduates is erroneous. A few procedures performed in OHNS; tracheotomy, rigid bronchoscopy, and rigid esophagoscopy to name a few, can be lifesaving, and despite low volume in the early years of practice, a high level of competence should be achieved during training. Exposure to these procedures is often achieved on rotations with a high clinical volume in under-represented OHNS subspecialties. Given the rarity of these procedures, a reasonable approach may include a periodic retraining or recertification requirement. In Ontario, head and neck cancer care is highly regionalized to high volume academic centers. [9] As such, graduates from OHNS are not required to have comprehensive surgical training in the surgical management of ablative mucosal procedures. However, parotidectomy and thyroidectomy, were frequently performed by recent graduates. The management of parotid and thyroid lesions sometimes necessitates a neck dissection, which was surprisingly the most common head and neck procedure performed in our cohort. Also, competence with neck dissection is important in the management of other life threatening OHNS emergencies such as incision and drainage of a deep neck space abscess and in rare cases being able to identify and ligate the external carotid artery in the management of severe epistaxis or tonsillar hemorrhage. Certainly, training in head and neck oncology is important for recent graduates, but the duration of surgical exposure to achieve competence has yet to be studied. Nonetheless, future work should assess whether there is ‘fee code’ creep towards procedures that provide increased compensation. The neck dissection code (R915) did experience a fee increase during the study years and future work should assess whether increase in fee code compensation impacts likelihood of generalists billing such codes.

In contrast, although otology demonstrated a very high rate of consultation and procedural codes, none of the procedural codes required exposure to advanced otologic procedures such as mastoidectomy, stapedotomy/stapedectomy, or even tympanoplasty (beyond type ones and myringoplasty). Nonetheless, a thorough understanding of middle and inner ear anatomy is often best achieved in the operating room, and the performance of advanced procedures such as cochlear implantation, provides an excellent model for residents to operate on ears without disease. This raises many important questions. It may be that recent OHNS graduates have insufficient surgical otology training during residency, the case volume as a generalist may be limited, and/or advanced otologic surgery may be regionalized in Ontario to high volume centers with fellowship trained otologists. Clearly, this requires further study and our results provide some of the preliminary results to help guide future work.

Our study has a number of strengths. In a single payer system, a complete data set of all billing codes for all new billing numbers in our specialty is obtainable. This is the first study of its kind in the medical literature and can be replicated in other jurisdictions. Our data abstraction was robust at delineating subspecialty diagnosis and billing codes given the use of experienced billers who are medical educators (P.C. and I.W.) and who are or have been program directors.

These data must be interpreted in the context of the study design. The most important limitation of this study is that it under-represents particular subspecialties, such as pediatrics and facial plastic surgery. Pediatric procedures, despite the lack of age data, were categorized under general (e.g. M&T and T&A), providing some insight into the proportion of procedures performed in this domain. Nearly all of the facial plastic surgery work is not billed under OHIP. This needs to be weighed into training curricula given the increase in facial cosmetic surgeons in Ontario and the increasing interest among OHNS generalists for performing non-surgical cosmetic injections (i.e. fillers and Botox). The data presented also does not account for the type of practice in which a new graduate begins independent practice which can heavily impact referral patterns, availability of operative time, and whether a new graduate is in a medical otolaryngology practice. Future work should address these specific concerns separately and look at a more recent cohort of graduates given the recent challenges with under employment among OTOHNS in the province. Another weakness is that the ICD-9 codes used in the billing database are not a perfectly reliable way of determining subspecialty consultation. Nonetheless, the data provides us with an approximation of what subspecialties the consultations fall within. Furthermore, it is unlikely that codes in one subspecialty belong in another and therefore our subspecialty level analysis is quite robust. Even a 5–10% change in the overall subspecialty proportion would not significantly impact the conclusions of our study. Future work should consider corroborating our findings with a cross sectional chart review of recent OTOHNS graduates. Lastly, the data provided was based on ‘new’ OHIP billing numbers but we could not with a high degree of certainty ascertain that all of the physicians identified were truly recent graduates. However, our exclusion of subspecialists makes our data more robust in that it excludes physicians who recently moved from other jurisdictions to develop a subspecialist/academic practice.

Future studies should compare these results to the training being offered to residents and shape future training to reflect the realities of the non-subspecialist practice in Ontario. Unfortunately, a standardized clinical log (with minimum case volumes) has not yet been adopted in Canada. Standardized case logging systems with minimum volumes and submission requirement for certification, should be considered in Canada. This would at least provide more consistent data collection. The role of simulation and boot camps have been fully incorporated into many Canadian OHNS programs and should be considered by all in the new CBD curriculum. [10–12] Procedure-specific evaluations will need to be developed for key indicator procedures [13] and this process has already begun (e.g. M&T). [14] More philosophically, narrowing the training of OHNS graduates to only the most commonly performed procedures may lead to other healthcare practitioners ultimately managing particular components of our practice (speech language pathologists, audiologists, nurse practitioners, physician assistants) all of whom can be trained to perform a very narrow component of our broad specialty. This has already started happening in the U.S. with mid-level practitioners decreasing the necessity for specialists and sub-specialists and ultimately decreasing their workload. [15–17] Similarly, if less residents are to rotate at certain hospitals and on certain rotations, for instance advanced head and neck oncology and advanced pediatric rotations, there will be a need for advanced practice providers to help manage the service and decrease resident service burden. [18]

## Conclusion

In conclusion, this is the first study exploring the scope of practice of newly employed otolaryngologist-head & neck surgeons using universal health care administrative billing data. This study demonstrates a high degree of otology and general otolaryngology volumes in consultations and a high degree of otologic, general, head and neck, and rhinological procedure volumes. These findings have important implications for future training and the development of CBD.

## Abbreviations

ABO: American Board of Otolaryngology
CBD: Competency based training otolaryngology-head & neck surgery
CBME: Competency based medical education
EPA: Entrustable professional activity
M&T: Myringotomy and tube insertion
MOHLTC: Ministry of health and long-term care
OHNS: Otolaryngology-head & neck surgery
RCPSC: Royal College of Physicians and Surgeons of Canada
T&A: Tonsillectomy and adenoidectomy
